# Complete mitochondrial genome of Siberian musk deer *Moschus moschiferus* (Artiodactyla: Moschidae) and phylogenetic relationship with other *moschus* species

**DOI:** 10.1080/23802359.2017.1407715

**Published:** 2017-11-25

**Authors:** Sang-In Kim, Mu-Yeong Lee, Hey Sook Jeon, Sang-Hoon Han, Junghwa An

**Affiliations:** a School of Earth and Environmental Sciences, Seoul National University, Seoul, Republic of Korea;; b Animal Resources Division, National Institute of Biological Resources, Incheon, Republic of Korea

**Keywords:** Siberian musk deer, *Moschus moschiferus*, genome, mitochondrion

## Abstract

The Siberian musk deer, *Moschus moschiferus*, is an Endangered species in South Korea due to its decreasing population size caused by illegal hunting and habitat destruction. In this study, the complete mitochondrial genome of *M. moschiferus* was determined using next-generation sequencing. Total length of its mitogenome is 16,356 bp in length, encoding 13 protein-coding genes, 22 transfer RNA genes, two ribosomal RNA genes, and one control region. Its AT contents are 62.4%, which are higher than its GC contents (37.7%) (A, 34.1%; C, 24.9%; G, 12.8%; and T, 28.3%). Phylogenetic relationship of genus *Moschus* showed topologies similar to those reported in previous studies. Sequence comparison between two *M. moschiferus* from South Korea indicated high sequence variations with 122 nucleotide differences. These results provide useful information necessary for further phylogenetic studies of *Moschus* species.

Siberian musk deer *Moschus moschiferus* is a species in genus *Moschus*, small deer-like ruminants have primitive features (Sathyakumar et al. 1993). Although the number of species in genus *Moschus* is controversial, seven species have been described: *M. moschiferus*, *M. chrysogaster*, *M. berezovskii*, *M. anhuiensis, M. leucogaster*, *M. fuscus*, and *M. cupreus* (Wilson and Mittermeier 2011). Among them, *M. moschiferus* occurs widely in Korea, Mongolia, Russia, China, and Kazakhstan (Tsendjav 2002). This species is classified as Vulnerable on the IUCN red list due to its continuously decreasing population caused by illegal hunting for musk and substantial habitat loss (Kang and Phipps 2003; Homes 2004; Nyambayar et al. 2015). Woo (1990) reported that the population size in South Korea is fewer than 40 individuals. To conserve this species, it is designated as a Class I Endangered species by Korean Ministry of Environment and a Natural Monument (No. 216) by Cultural Heritage Administration of Korea. In this study, we described the mitogenome of *M. moschiferus* using next-generation sequencing (NGS) to provide basic genetic information about this species.

A specimen (IN156) of *M. moschiferus* was collected from Hwacheon-gun, Gangwon-do, South Korea after obtaining permit of related regulation (from the Cultural Heritage Administration of Korea). It was deposited at National Institute of Biological Resources at Incheon, South Korea. Total genomic DNA was isolated from a muscle tissue sample using Genome Wizard Kit (Promega, Madison, WI) according to the manufacture’s instruction. Genomic DNA isolated from the sample was then sequenced using NGS (Hahn et al. 2013). NEXTflex™ Rapid DNA-Seq (Bioo Scientific, Austin, TX) and Accel-NGS +2 PCR free kit (Swift Bioscience, Ann Arbor, MI) were used to prepare sequencing libraries. The circular mitogenome of *M. moschiferus* is 16,356 bp in length, encoding 13 protein-coding genes, 22 transfer RNA genes, two ribosomal RNA genes, and one putative control region (D-loop region). A tandem repeat was not found in the D-loop region. Overall base compositions for A, C, G, and T in this mitogenome were 34.1, 24.9, 12.8, and 28.3%, respectively, with AT contents of 62.4%. Similar to typical vertebrate mitogenome, all genes in mitogenome of *M. moschiferus* are distributed in the H-strand except *ND6* subunit gene and eight tRNAs that are encoded on the L-strand. The mitogenome of *M. moschiferus* generated in this study was deposited in GenBank under accession number KT337321.

For phylogenetic analyses of genus *Moschus*, 13 protein-coding genes from seven complete mitogenomes were retrieved from GenBank (Peng et al. 2009; Jang and Hwang 2010; Hassanin et al. 2012; Wang et al. 2013; Zhu et al. 2013; Pan et al. 2015) and edited with Geneious Pro v11.0.2 (Biomatters; Kearse et al. 2012). MUSCLE (Edgar 2004) was used for whole-genome alignment. Phylogenetic tree (Figure 1) was reconstructed using Neighbour-Joining (NJ) method by MEGA 6 (Tamura et al. 2013). Phylogenetic relationships among four species of genus *Moschus* were consistent with the monophyletic pattern reported previously (Pan et al. 2015). Three haplotypes of *M. moschiferus* clustered in one clade. Interestingly, *M. moschiferus* (KT337321) in this study was remotely related to other sequences of *M. moschiferus* (FJ469675 and JN632662). A total of 122 and 102 of nucleotide differences were observed between KT337321 and other two haplotypes of *M. moschiferus,* FJ469675 and JN632662, respectively. This sequence comparison indicated relatively larger genetic polymorphism within *M. moschiferus* than that within other *Moschus* species (32 for *M. chrysogaster* and 18 for *M. anhuiensis*). These results will contribute to improve our understanding of phylogenetic relationships of genus *Moschus* species and subspecies of *M. moschiferus*.

**Figure 1.**
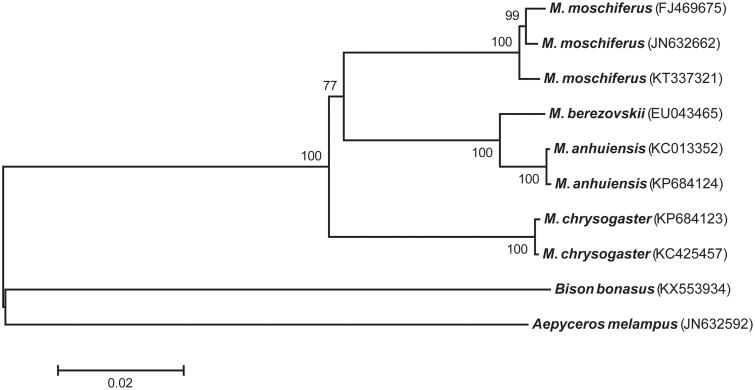
Phylogenetic tree for four species of Genus *Moschus* based on concatenated nucleotide sequences of 13 protein-coding genes. Bootstrap replicates were performed 5000 times. Numbers on nodes indicate bootstrap value. GenBank accession number of each species used for tree construction is listed after the species name. *Aepyceros melampus* (JN632592) and *Bison bonasus* (KX553934) were used as out-group species.
